# Supplementary material for: The interactome of intact mitochondria by cross-linking mass spectrometry provides evidence for coexisting respiratory supercomplexes
Source: Mol Cell Proteomics. 2017 Dec 8;17(2):216–32. doi: 10.1074/mcp.RA117.000470 (PMC5795388; doi:10.1074/mcp.RA117.000470)
Supplement: Supplemental Data [file supp_RA117.000470_133922_0_supp_23978_fzffwf.zip › spectra_annotation/mito_spectra_annotation/285-1-4-1-3-1.pdf]

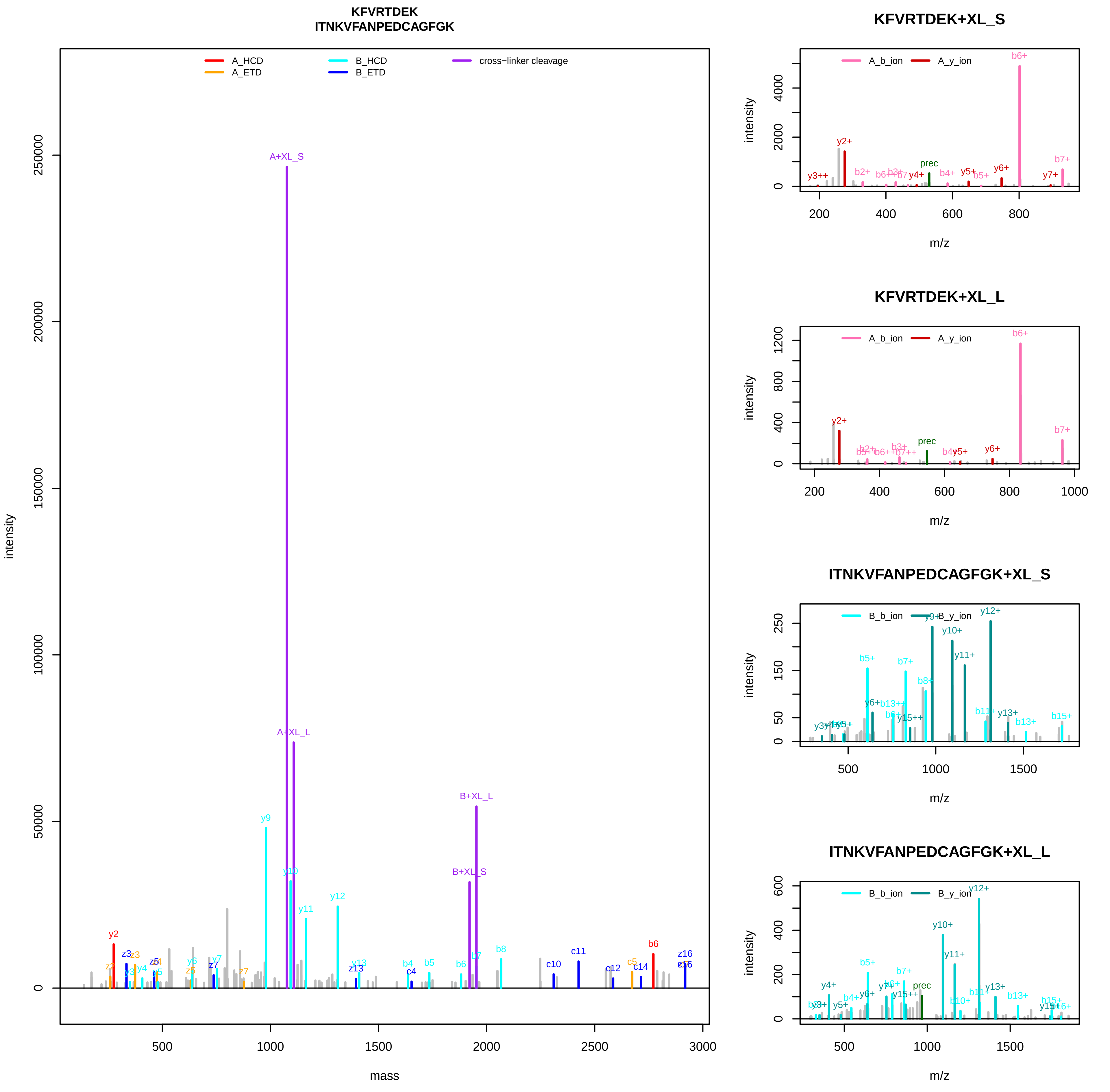

KFVRTDEK
ITNKVFANPEDCAGFGK
A_HCD
A_ETD
B_HCD
B_ETD
cross−linker cleavage
A+XL_S
A+XL_L
B+XL_L
B+XL_S
intensity
mass
0
50000
100000
150000
200000
250000
500
1000
1500
2000
2500
3000
y2
y9
y10
y11
y12
y13
z13
b4
c4
b5
b6
b7
b8
c10
c11
c12
c14
c5
b6
z16
KFVRTDEK+XL_S
A_b_ion
A_y_ion
b6+
y2+
prec
b7+
y7+
y6+
y5+
b5+
b4+
y4+
y3++
b2+
m/z
KFVRTDEK+XL_L
ITNKVFANPEDCAGFGK+XL_S
B_b_ion
B_y_ion
y12+
y10+
y11+
y9+
b5+
b7+
b8+
y13+
b13+
b15+
ITNKVFANPEDCAGFGK+XL_L
y4+
y5+
b10+
b11+
b15+
